# Separation and recovery of carbon powder in anodes from spent lithium-ion batteries to synthesize graphene

**DOI:** 10.1038/s41598-019-46393-4

**Published:** 2019-07-08

**Authors:** Li Yang, Liu Yang, Guangri Xu, Qigao Feng, Yuanchao Li, Erqing Zhao, Jingjing Ma, Shumin Fan, Xiaobo Li

**Affiliations:** Chemistry and Chemical Engineering, Henan Institute of Science and Technology, Xinxiang, Henan 453003 P.R. China

**Keywords:** Environmental impact, Carbon capture and storage

## Abstract

Based on the structural characteristics of the anodes of lithium-ion batteries, an improved Hummers’ method is proposed to recycle the anode materials of spent lithium-ion batteries into graphene. In order to effectively separate the active material from the copper foil, water was selected as an ultrasonic solvent in this experiment. In order to further verify whether lithium ions exist in the active material, carbon powder, it was digested by microwave digestion. ICP-AES was then used to analyse the solution. It was found that lithium ions were almost non-existent in the carbon powder. In order to further increase the added value of the active material, graphene oxide was obtained by an improved Hummers’ method using the carbon powder. The graphene material was also reduced by adding vitamin C as a reducing agent through a chemical reduction method using graphene oxide. Meanwhile, the negative graphite, graphite oxide and graphene samples were characterized by XRD, SEM, FTIR and TEM. The conductivity of the negative graphite, graphite oxide and graphene was tested. The results show that graphene prepared by a redox method has a better layered structure, less impurities and oxygen groups in its molecular structure, wider interlayer spacing and smaller resistivity.

## Introduction

Lithium-ion batteries have become ideal energy sources in the 21st century due to their lightweight, small volume, high specific energy, small self-discharge and long cycle life^1–4^. Because silicon-based anodes have huge reserves and super high theoretical capacity (4200 mAh g^−1^ for Li_4.4_Si), which is approximately 10 times higher than the state-of-the-art graphitic anode (372 mAh g^−1^ for LiC_6_), Silicon (Si) has been considered as one of the most promising anode material for the next generation lithium-ion batteries (LIBs)^5,6^. With the continuous development and application of silicon-based lithium-ion batteries, all-solid-state lithium-ion batteries, aluminum-air batteries and other batteries, the output of batteries has increased year after year^7–10^. In addition, the total quantity and weight of these exhausted batteries in China will exceed 25 billion units and 500,000 tons in 2020, as surveyed by Zeng *et al*.^11^. The recovery and treatment of spent lithium-ion batteries mainly concentrate on the separation, recovery and refining of cobalt and lithium as cathode and precious metals. However, little research has been done on the separation, recovery and recycling of anode active materials^12,13^. Spent lithium-ion battery anodes that have not been reasonably recycled lead to the release of pollutants into the natural environment, which may threaten human health. However, the carbon powder adhering to copper foil in the anode electrode of spent lithium-ion batteries has very large utilization potential and high recovery value. Therefore, the effective separation of the anode electrode components of spent lithium-ion batteries would play an important role in maximizing their resource utilization and eliminating the corresponding impact on the environment^14,15^. In order to alleviate the serious resource shortage and environmental pollution caused by rapid economic development, it has become a global consensus to recycle all components of spent lithium-ion batteries.

The recovery of the anodes of spent lithium-ion batteries is mainly concentrated on copper and carbon powder. Copper (~35%) separated from the anodes of spent lithium-ion batteries can be reused directly. It is an important raw material widely used in production. However, most of the carbon powder adhering to it (~60% content) has been abandoned and has not been rationally utilized. Usually, there are also some methods concentrating on the recovery of anode electrodes in combination with three different concepts. The first method is based on a separation or leaching process in order to recovery copper foil and lithium^16,17^. The second method based on a thermal treatment of graphite to recycle and reuse as high-performance cathode^18^. And the final investigated approach uses a liquid carbon dioxide or supercritical carbon dioxide as extractant to recycle electrolyte^19^.

In recent years, several traditional methods for recycling spent lithium-ion batteries have been developed, including hydrometallurgy^20–23^, pyrometallurgy^24^ and mechanical physics^25,26^. However, most of these methods are complex, time-consuming and expensive to operate. The redox method forconverting carbon powder into graphene proposed in this study is expected to be a more suitable alternative. The method has the mainadvantages of simple operation, low costand high yield. Furthermore, themethod also shows that the great potential ofrecycling spent battery anode materials.

Graphene is a single layer of carbon atom surface material, which is stripped from graphite. It is a two-dimensional structure of carbon and is deemed a “super material”. Its hardness is greater than that of diamond, and it has the same elasticityas rubber. Its electric and thermal conductivity are higher than copper, and its weight is almost negligible compared with copper. A crystalline film of graphene is only 0.335 nm thick. The discovery of graphene was a landmark in nanotechnology. Graphene has excellent properties in optics, electricity, mechanics, thermology and other fields. Graphene has many advantages in nano-electronic devices, highly sensitive sensors, transparent conductive films, functional composites, energy storage, catalysis and other fields. Its unique atomic structure, high electrical conductivity^27^, high elastic modulus, low thermal expansion coefficient and high thermal fatigue and creep resistance are considered to be revolutionary for materials in the future^28–30^.

Graphene has unique physical and chemical properties. The main preparation methods are mechanical stripping^31^, chemical vapor deposition^32^, epitaxial growth^33^ and oxidation-reduction^34^. Through consulting a large number of literature studies, it is found that the graphene oxide reduction method is easier to operate than the other methods. The production cost is greatly reduced, graphene can be prepared on a large scale, and functional graphene oxide can be prepared while graphene is prepared. Most carbon powders in the anode electrodes of spent lithium-ion batteries have been discarded, and have not been rationally recycled. This not only wastes carbon resources, but also causes environmental pollution. Here, for the first time, a new method is proposed to prepare graphene using an oxidation-reduction method to recover the anode material of spent lithium-ion batteries. The structures of the products were analyzed by X-ray diffraction (XRD), scanning electron microscopy (SEM), transmission electron microscopy (TEM) and Fourier transform infrared spectroscopy (FTIR). This research not only focuses on the serious problems of resource shortages and environmental pollution caused by rapid economic development, but also maximizes the resource utilization of spent lithium-ion batteries.

## Experimental

The spent lithium-ion batteries needed for the experiment were collected from various places, and the rated capacity ranged from 550 to 1250 mA·H. The spent lithium-ion batteries were mechanically disassembled and the cathode, anode and diaphragm of the batteries were separated. The anode material was collected and cut it into 1 cm × 5 cm pieces for use. Concentrated phosphoric acid, hydrochloric acid (5%), N-methyl-2-pyrrolidone (NMP) and acetone were purchased from the Tianda Chemical Reagent Factory (Dongli District, Tianjin, China). Ethylene glycol, concentrated sulfuric acid, potassium permanganate, ammonia water, hydrogen peroxide and vitamin C were purchased from the Shanghai National Pharmaceutical Reagent Factory (Huangpu District, Shanghai, China). All the above reagents were analytically pure in this experiment. An ultrapure water system (SG Ultraclear system, Wasseraufbereitung und Regenerierstation GmbH, Hamburg, Germany) was used to produce the ultrasonic solvent with a specific conductivity of 0.055 μS/cm.

### Recycling of anode materials

First, the packaging and shell of the spent lithium-ion batteries were peeled off by means of mechanical tools, and the cores were taken out. The cathode and anode materials were then separated. The anode material was cut into small pieces of 1 cm × 5 cm, and the carbon in the anode material was separated from the copper foil by an ultrasonic leaching method. Ultrasound leaching is an environmentally-friendly and efficient method for the separation of copper and carbon powder, because it does not require chemical reagents, only uses water and has low energy consumption. A quantity of 0.25 g of anode material was weighed and put it in an electrothermal drying chamber at a constant temperature of 120 °C for 1 h. After cooling, the cathode material and copper foil were separated completely at normal room temperature after 2 min of ultrasound treatment. The separated copper foil and carbon powder were dried in an electrothermal drying oven at a constant temperature of 120 °C for 1 h. The carbon powder content in the anode material was calculated as 77%. The separation efficiency of the active substances in the anode material was calculated as follows (1):

1 $${\rm{Separation}}\,{\rm{efficiency}}\,( \% )=\frac{{\rm{active}}\,{\rm{substance}}\,{\rm{of}}\,{\rm{anode}}\,\mathrm{material}\,}{{\rm{anode}}\,{\rm{material}}\,{\rm{with}}\,{\rm{copper}}\,{\rm{foil}}}\times 100 \% $$

### Preparation of graphene oxide

Concentrated sulfuric acid and phosphoric acid with a volume ratio of 9:1 and 1 g of carbon powder were put into a small beaker, and then 6 g of potassium permanganate was added in succession to prevent a violent reaction. The solution was stirred in an iced water bath for 1 h, then placed into a small beaker, whereupon the solution was stirred and maintained at a temperature of 50 °C and reacted at this temperature for 12 h. Iced water was added to the product, and hydrogen peroxide (30% volume fraction) was added dropwise while stirring until the color of the solution turned golden. Then, the centrifugation was started, and the centrifugation time was 4 000 r min^−1^ for 3 min. The lower solids of the centrifugal tube were taken, and the products were washed with hydrochloric acid (5% volume fraction) and distilled water until the pH was ~7. Finally, the obtained graphite oxide was dispersed in water, then the graphite oxide was ultrasonically cleaned for 8 h with an ultrasonic cleaner, placed in a vacuum drying box and dried for later use.

### Preparation of grapheme

**Figure 1:**
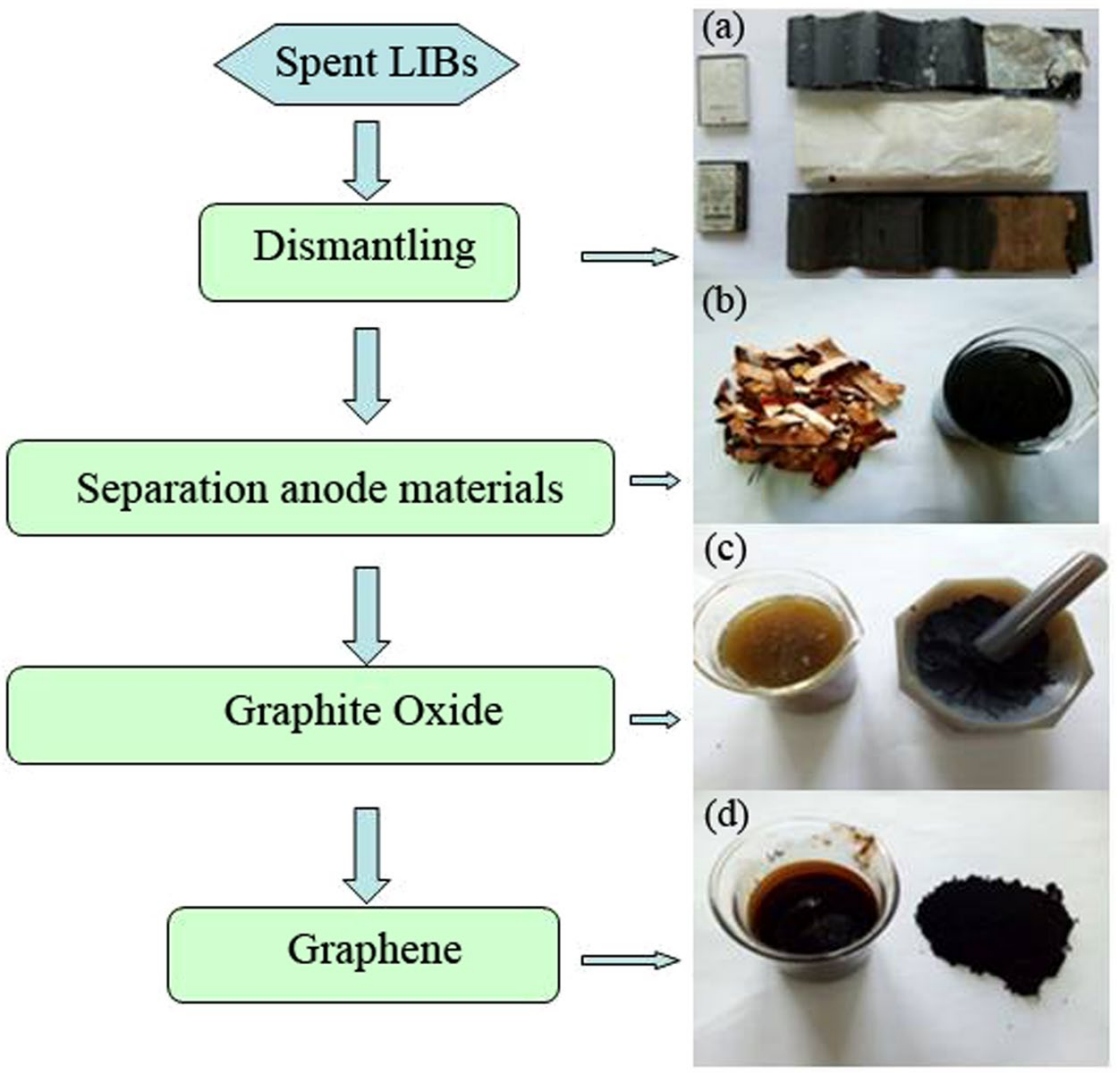
Preparation process of graphene production from spent lithium-ion batteries.

A stable graphite oxide dispersion solution can be obtained by dispersing 10 mg of graphite oxide in a 25 ml aqueous solution, and then ultrasonically dispersing for 1 h until it becomes a brown suspension. Graphene oxide dispersions were moved into a 100 ml beaker and ammonia water was added to the beaker to make the pH ~8. After that, 250 mg of vitamin C was added to the reaction solution, stirred for 0.5 h, then the homogeneous solution was placed in a magnetic stirrer and stirred at 90 °C for 1 h. Next, the stable black dispersions were dried in a vacuum drying chamber at 60 °C for 48 h, and the graphene samples were finally obtained. After the sample was fully dried, the agate mortar was used to grind the powder for reserve. As shown in Fig. 1, the preparation process of graphene production from spent lithium-ion batteries is as follows (Fig. 1).

### Material characterization

The samples were analyzed by a Tensor 27 Fourier transform infrared spectrometer using the KBr pressing method, with a scanning wavenumber ranging from 400 to 4000 cm^−1^. Diffraction patterns were collected using an Advance D8 X-ray diffractometer with a scanning range of 10–80°. The appearance and internal morphology of the samples were analyzed with a Quanta 200 scanning electron microscope and Hitachi HT7700 transmission electron microscope. The resistivity of the samples was measured by a RTS-9 four-probe tester.

## Results and Discussion

### Optimum separation conditions of active material and copper foil

In order to obtain the optimal conditions for the separation of anode materials, NMP, H_2_O, acetone and ethaneiol were used as ultrasonic solvents, which could be used to separate the anode materials efficiently during the course of the experiment. Table 1 shows that water is the best solvent in the process of ultrasonic extraction, while NMP is the highest of the other three solvents. Therefore, this experiment shows that water plays a very important role in the separation of active substances from copper foil. From the aspect of the leaching mechanism, ultrasound provides significant energy for the leaching process and improves the leaching efficiency. In addition, cavitation occurs in the liquid under the action of ultrasound, which causes insoluble substances, such as poly (vinylidene fluoride) to covered on the surface of the anode material and separate from the copper foil and form a highly active surface. Moreover, the large amount of energy released during the whole process can help the separation of anode active substances and copper foil effectively. Finally, water was chosen as the ultrasonic solvent, and the active material of the anode material could be separated from the copper foil by ultrasound at room temperature for 2 min. The copper foil was restored to its original metal form.

**Table 1:** Solubility of different solvents.

Leaching solvent	NMP	H_2_O	Acetone	Ethanediol
Molecular formula	C_5_H_9_NO	H_2_O	CH_3_COCH_3_	C_2_H_6_O_2_
Relative molecular mass	99.13	18.02	58.08	62.07
Solubility/(g/L, 20 °C)	soluble	soluble	soluble	soluble
Density/(g/cm^3^)	1.026	1	0.788	1.1155
Boiling point/(20 °C)	203	100	56.53	197.3
Dissolution rate/%	57.4	99.5	49.6	30.8

### FTIR characterization of acetylene black, the cathode of graphite, graphite oxide and grapheme

**Figure 2:**
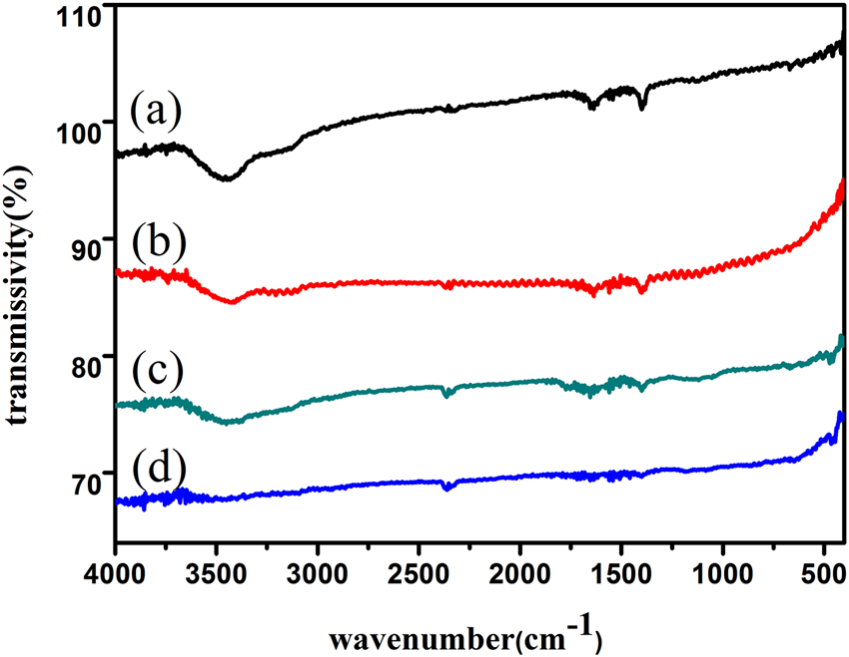
FTIR spectra of (**a**) acetylene black, (**b**) the cathode of graphite, (**c**) graphite oxide and (**d**) graphene.

It can be seen from Fig. 2(a,b) that there is an absorption peak at 2356 cm^−1^, which belongs to the stretching vibration absorption peak of the C=C bond in the sp^2^ structure of the graphite crystal^35^. In the high frequency region at ~3430 cm^−1^, it belongs to the stretching vibration of OH. The wider spectrum peaks in the range of 3000–3700 cm^−1^ comes from the water molecules (Fig. 2(c)). Because of the strong hygroscopicity of the sample, the absorption peak corresponding to the bending vibration of OH at 1635 cm^−1^ is stronger, and the stretching vibration of water molecules at 3200–3700 cm^−1^ leads to the broadening of the spectral peak. This indicates that the water molecules are difficult to remove (Fig. 2(c)). The absorption peak at 1720 cm^−1^ belongs to the stretching vibration peak of C=O, and the peak at 1265 cm^−1^ belongs to the vibration absorption peak of C-O-C (Fig. 2(c)). This shows that graphite oxide under this experimental condition contains at least three functional groups: OH, C=O and C-O-C. The presence of these oxygen-containing groups indicates that graphite has been oxidized. When graphite oxide is reduced by vitamin C, a relatively weak and narrow absorption peak appears at 3350 cm^−1^ (Fig. 2(c)), which may be caused by a small amount of unreduced OH and adsorbed water molecules. The FTIR spectra of graphene reduced by vitamin C (Fig. 2(d)) are very similar to those of acetylene black and the cathode of graphite (Fig. 2(a,b), respectively) which indicates that they are all one kind of carbon. Compared with the FTIR spectra ofgraphite oxide (Fig. 2(c)), the absorption peaks of graphene (Fig. 2(d)) are reduced by vitamin C, and the absorption peaks caused by the vibration of surface functional groups almost disappear, indicating that the oxygen-containing groups were essentially removed (Fig. 2(d)).

### XRD characterization of acetylene black, the cathode of graphite, graphite oxide and grapheme

**Figure 3:**
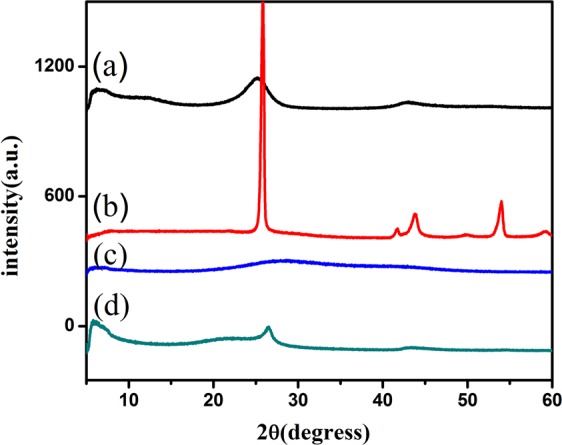
XRD patterns of (**a**) acetylene black, (**b**) anode graphite, (**c**) graphite oxide and (**d**) graphene.

XRD is mainly used to characterize the crystal structure, crystal plane spacing, lattice parameters and crystallinity of materials. The restore degree, interlayer spacing and defects of graphene can be analyzed and evaluated. Figure 3(a,b) showsharp diffraction peaks near 2θ of ~26.5°, which indicate that graphite has a very high degree of crystallization and the spatial arrangement of the crystal sheets is very regular. After graphite is oxidized, a weaker and wider diffraction peak appears near 2θ of ~10.6°, which is the diffraction peak of graphite oxide (Fig. 3(c)). This indicates that the crystal form of graphite is destroyed and a new crystal structure is formed. When graphite oxide is reduced to graphene, the diffraction peak of graphene appears near 2θ of ~26° (Fig. 3(d)), which is similar to the position of the diffraction peak of anode graphite (Fig. 3(b)), but the diffraction peak broadens and the intensity decreases. This is due to the reduction of graphite lamellae, the decrease of crystal structure integrity and the increase of disorder.

### SEM of the cathode of graphite, graphite oxide and graphene and TEM of grapheme

**Figure 4:**
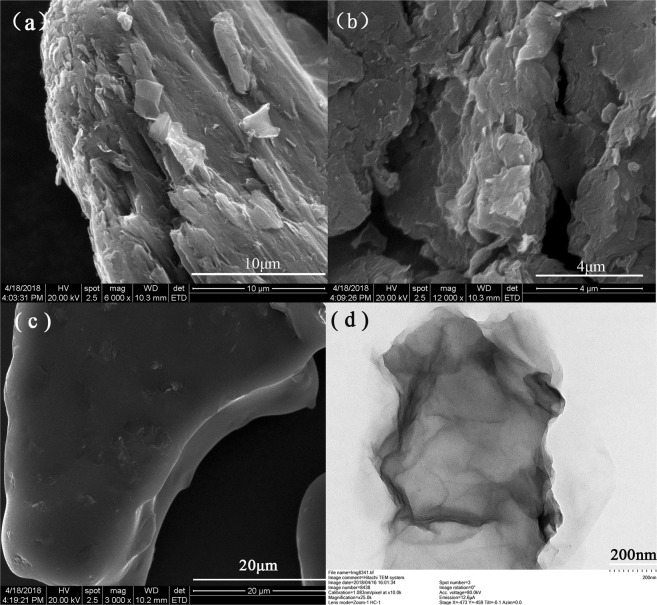
SEM images of (**a**) the cathode of graphite, (**b**) graphite oxide, (**c**) graphene and (**d**) TEM image of graphene.

SEM is usually used to observe and analyze the surface morphology of graphite, graphite oxide and various graphene materials. It can be seen that graphene presents an obvious accumulation state, folding phenomenon is obvious, the distance between layers increases, and only at the edge of the layers has obvious layered structure (Fig. 4(c)). Because the nanoparticles have strong aggregation characteristics, it is difficult to see the thin yarn layered structure of graphene in the scanning electron microscope (Fig. 4(c)). Figure 4(d) shows TEM image of graphene reduced by vitamin C with different magnifications. The surface morphology of graphene can be observed by TEM. It can be seen from the image that graphene shows a transparent sheet structure under. The thickness of the graphene sample is very thin and folds can be observed in some areas of the sample, which is due to the overlapping of graphene sheets or the curling of edge areas. From the high-resolution image, we can see that the surface texture of graphene is obviously smooth and ordered. With the increase of magnification factor, the thin yarn layered structure of graphite becomes more and more obvious.

### Resistivity of acetylene black, graphite oxide and grapheme

Minimal resistivity is a wonderful feature of graphene. Graphene was prepared by an improved Hummers method. During the oxidation process, the conjugated structure of graphite sheet was destroyed. A large number of oxygen-containing groups (hydroxyl, carboxyl and epoxy) were grafted onto the surface of the graphene sheet, and the resistivity increased. However, after vitamin C reduction, a large number of oxygen-containing groups on the graphene surface were removed, and the sheets were opened in a large area. The structure of graphene was restored and the conductivity was improved. The resistivity of acetylene black (d = 1 cm), graphite oxide (d = 1 cm) and graphene (d = 1 cm) were measured by a four-probe tester. Three areas were selected for the surface measurement of the sample film, as shown in Table 2. However, the resistivity is still lower than the theoretical value, which indicates that some oxygen-containing groups have not been removed during the reduction process, resulting in defects in the results and poor conductivity.

**Table 2:** Resistance of acetylene black, graphite oxide and graphene.

Sample	Resistivity/(Ω · m)
Acetylene black	89
Graphite oxide	451.2
Graphene	41.6

## Conclusion

The potential value of spent lithium-ion battery anode materials is constantly being explored. In this experiment, the ultrasonic leaching method was used. After optimizing the experimental conditions, water was chosen as the ultrasonic solvent to recover carbon powder from the negative material of spent lithium-ion batteries. The improved Hummers’ method was used to convert carbon powder into graphite oxide. Finally, graphene was prepared with vitamin C as a reducing agent. XRD analysis showed that most of the oxygen-containing functional groups of graphene were removed by a reduction reaction. FTIR characterization showed that a large number of oxygen-containing groups on the surface of graphene were removed, the structure of graphene was restored, and the reducibility of vitamin C was very good. Graphene was observed by SEM and TEM. The obvious layered structure of graphene was observed, which proved that graphene was obtained. The resistivity of graphene was measured by a four-probe tester. It was shown that the oxygen-containing groups on the surface of graphene were largely removed and the structure of graphene was restored. Moreover, the whole process cost is very low, which provides a new method and way for the recovery and utilization of spent lithium-ion batteries.
